# The widespread presence of non-nutritive sweeteners challenges adherence to beverage guidance for children

**DOI:** 10.3389/fpubh.2023.1221764

**Published:** 2023-08-16

**Authors:** Mariana Fagundes Grilo, Lindsey Smith Taillie, Allison C. Sylvetsky

**Affiliations:** ^1^Department of Exercise and Nutrition Sciences, Milken Institute School of Public Health, The George Washington University, Washington, DC, United States; ^2^Sumner M. Redstone Global Center for Prevention and Wellness, The George Washington University, Washington, DC, United States; ^3^Carolina Population Center, University of North Carolina at Chapel Hill, Chapel Hill, NC, United States

**Keywords:** artificial sweeteners, sugar substitutes, low-calorie sweeteners, non-nutritive sweeteners, beverages, children, nutritional labeling, front-of-package (FOP) labels

## Introduction

Non-nutritive sweeteners (NNS), including aspartame, sucralose, saccharin, acesulfame-K, neotame and advantame are food additives approved for use in foods and beverages by the US Food and Drug Administration (FDA) (1). Two additional NNS, steviol glycosides (often referred to as “stevia”) and monk fruit, are considered to have GRAS (“Generally Recognized as Safe”) status and are permitted for specific conditions of use in the food supply (2). NNS are a heterogeneous group of compounds with different chemical structures, however they are all potently sweet relative to sugar and contain no or few calories (1), which makes them popular substitutes for added sugars in foods and beverages (3).

1. Role of NNS intake in weight management and chronic disease

While NNS are believed to be safe for human consumption (1, 2), there is continued controversy regarding their role in weight management and cardiometabolic disease prevention (4–6). Among adults, findings of randomized controlled trials (RCTs) in which NNS are used to replace added sugars demonstrate that NNS may favor weight reduction and maintenance, especially when used as part of structured diet plans (7).

However, findings of recent prospective cohort studies and small RCTs have demonstrated potentially harmful metabolic and health effects associated with the consumption of NNS (8, 9). Consumption of NNS (typically assessed as a group or using diet soda as a proxy) has been reported to increase the risk of cardiovascular disease, cancer, and all-cause mortality (10). Unfavorable metabolic effects of certain types of NNS have also been reported. For example, chronic consumption of sucralose has been shown to affect insulin and glucose responses in non-insulin resistant adults (11). Ingestion of acesulfame-K and saccharin can affect the composition of the gut microbiota, resulting in glucose resistance (12).

2. Research on NNS consumption in children is lacking

Research examining effects of NNS intake in children is particularly scarce (13) and widespread consumption of NNS among children is concerning given the potential for adverse health effects reported in adults (7, 8). Studying the role of NNS in children's health is also important because eating habits and taste preferences are shaped during childhood and may persist into adulthood (14).

Meanwhile, foods and beverages containing NNS are increasingly marketed to and consumed by children (3, 15). While parents and caregivers (hereafter parents) express concern about the safety of NNS-containing beverages for children (16), they often do not recognize products with NNS (17); further, identifying NNS in foods and beverages is especially challenging for individuals with limited nutrition and health literacy, which may disproportionately promote inadvertent NNS consumption among children from disadvantaged backgrounds. In this opinion article, we highlight the importance of regulatory actions to support parents in identifying NNS in the food supply and emphasize the need to closely monitor the use of NNS, especially in beverages marketed to and consumed by children. Such actions are particularly urgent in light of ongoing policy efforts to reduce added sugar intake, which are likely to lead to continued increases in the use of NNS in the food supply (18).

3. Recommendations for NNS intake among children

Considering the dearth of evidence on the metabolic and health effects of NNS in children, the American Academy of Pediatrics (AAP) stated that more data on the health effects of NNS is needed. And in a recent policy, the APP also advocated for disclosing amounts of NNS per serving on food labels in order to more carefully monitor NNS intake among children (3). Due to the lack of available evidence on the potential adverse effects of consuming beverages with NNS relative to potential benefits, the American Heart Association (AHA) recommended against consumption of NNS by children in a 2018 Science Advisory and reinforced that potential benefits of replacing added sugars with NNS in decreasing total energy and aiding weight loss/weight control would not be fully realized if there is a compensatory increase in energy intake from other sources. Finally, the National Academy of Science Engineering and Medicine reviewed recommendations on NNS and highlighted the lack of consistency in current recommendations surrounding NNS intake among children (19).

4. Presence of NNS in beverages marketed to children

NNS are increasingly present in a wide variety of products marketed to children (3). Between 2009 and 2012, 21.5% of children between 2 and 5 years old consumed NNS from any source and 13.3% consumed NNS in beverages (15). Equally concerning is the fact that parents are often unable to recognize NNS in beverages marketed to children and inadvertently provide products with NNS to them (16, 20).

Beverages are a particularly problematic product category when it comes to NNS consumption among children. As of 2019, more than 70% of beverages marketed to children in the US contained NNS, and 40% contained both NNS and added sugars (3). NNS were found mostly in fruit drinks as well as in products marketed as “water beverages” (3). Yet, the majority of NNS-containing beverages marketed to children did not contain any front-of-package information indicating the presence of NNS, which makes it difficult for parents to recognize that these products contained NNS (3).

Furthermore, a study that assessed parents' perceptions of NNS in beverages found that less than one third of parents who provided NNS-containing fruit drinks or flavored waters to their child accurately identified the beverage as containing NNS (17). Meanwhile, parents reported concern with the safety of NNS-containing beverages for children (16, 17), which suggests that they would likely not provide these products to their children if the information about NNS was clearly disclosed on the package (17). In addition, the widespread use of product packaging that features pictures of fruit, and child-directed marketing, and nutrient content (e.g., sugar-free, 100% vitamin C) and/or ingredient claims (e.g., no high-fructose corn syrup) on the front-of-package, leads parents to mistakenly perceive products as healthy and encourages them to unknowingly purchase NNS-containing beverages for their children (20).

Because food labeling regulations in the US do not require front-of-package disclosure of NNS, identifying products with NNS requires that parents carefully read the ingredient lists, and be familiar with technical terms for NNS, which are listed in small letters on the back or side of the package. This makes recognition of products with NNS particularly challenging, especially for individuals with limited nutrition and health literacy, and may disproportionately promote inadvertent NNS consumption among children from disadvantaged backgrounds. In fact, a recent study in a virtual supermarket in the US showed that only 12% of parents of children aged between 1 and 5 years old checked the nutrition facts panel when selecting a snack for their child (21), further reiterating the need for clearer labeling on the front-of-package.

5. Difficulties in estimating exposure to NNS among children

While the consumption of NNS among children has increased in recent years (15), there are challenges in estimating exposure to NNS at the population level. Current levels of NNS exposure are underestimated for a variety of reasons, including the fact that most studies rely on self-report (or in the case of young children, parent-report) to assess NNS intake, which requires participants to accurately recall consumption of products with NNS. Further, food and nutrient databases often do not provide the specificity to accurately detect NNS presence and products are continually reformulated making it difficult for food and nutrient databases to reflect current product ingredient composition. Finally, multiple NNS are frequently used in combination (e.g., sucralose and acesulfame-potassium), which further complicates accurate quantification and monitoring of NNS intake.

## Discussion

Accumulating data demonstrating unfavorable health effects of NNS consumption in adults and the continued scarcity of available evidence in children underscore the need for a more conservative approach surrounding NNS consumption. Based on the precautionary principle (22), it is prudent to take action to limit exposure to NNS among children in order to reduce the potential for unintended adverse consequences, even though evidence of harm is not conclusive. For example, at the governmental level, use of NNS in beverages marketed to children should be regulated. Furthermore, manufacturers should be required to more clearly indicate that a product contains NNS in order to increase parents' ability to correctly identify NNS in products and make informed decisions about what to provide to their children. Mexico already requires a front-of-package warning label on products containing NNS, which reads “contains sweeteners, not recommended for children” (23). Colombia and Argentina also passed a law that requires a warning label on products containing NNS (24, 25). While front-of-package labels for NNS are a recent emergence, front-of-package labels for added sugars have been widely tested and shown to be effective for discouraging parents from purchasing unhealthy products for their children (21). Additional studies are needed to determine if similar effects would be observed for NNS labels, and how this information would be most effectively communicated to parents on product packaging.

The importance of supporting parents in identifying NNS in beverages is especially relevant when considering recent trends in children's NNS consumption observed in Latin American countries after implementation of public health policies to reduce added sugars in the food supply. For example, in Chile, where a front-of-package warning label has been adopted for added sugars but not for NNS, there has been an increase of the presence and consumption of NNS in beverages, yogurts, and other products consumed by children (18).

Product reformulation leading to increases in NNS use has consistently been observed following implementation of policies to reduce added sugar intake globally. Health authorities should therefore take regulatory actions to support parents in identifying NNS in food and beverage products and closely monitor the use of NNS, especially in beverages marketed to children. Furthermore, better alignment in messaging around NNS consumption between regulatory agencies and nutrition and health professionals is needed to support parents in making informed decisions about what products to provide their children (14). While different NNS have different chemical and physical properties and may have divergent effects on health, few studies have directly compared effects of different NNS on weight and metabolic outcomes, especially among children, and this represents an important area for future research.

## Author contributions

MFG and ACS designed the manuscript. MFG wrote the first draft of the manuscript. ACS and LST contributed to the writing and review of the manuscript. All authors contributed to the article and approved the submitted version.
